# Differential Sex-Dependent Regulation of the Alveolar Macrophage miRNome of SP-A2 and co-ex (SP-A1/SP-A2) and Sex Differences Attenuation after 18 h of Ozone Exposure

**DOI:** 10.3390/antiox9121190

**Published:** 2020-11-27

**Authors:** Nithyananda Thorenoor, David S. Phelps, Joanna Floros

**Affiliations:** 1 Center for Host Defense, Inflammation, and Lung Disease (CHILD) Research, Department of Pediatrics, College of Medicine, The Pennsylvania State University, Hershey, PA 17033, USA; nthorenoor@pennstatehealth.psu.edu (N.T.); dphelps@pennstatehealth.psu.edu (D.S.P.); 2 Department of Biochemistry & Molecular Biology, College of Medicine, The Pennsylvania State University, Hershey, PA 17033, USA; 3 Department of Obstetrics & Gynecology, College of Medicine, The Pennsylvania State University, Hershey, PA 17033, USA

**Keywords:** alveolar macrophages, miRNA, SP-A2, co-ex (SP-A1/SP-A2), surfactant

## Abstract

Background: Human SP-A1 and SP-A2, encoded by *SFTPA1* and *SFTPA2*, and their genetic variants differentially impact alveolar macrophage (AM) functions and regulation, including the miRNome. We investigated whether miRNome differences previously observed between AM from SP-A2 and SP-A1/SP-A2 mice are due to continued qualitative differences or a delayed response of mice carrying a single gene. Methods: Human transgenic (hTG) mice, carrying SP-A2 or both SP-A genes, and SP-A-KO mice were exposed to filtered air (FA) or ozone (O_3_)_._ AM miRNA levels, target gene expression, and pathways determined 18 h after O_3_ exposure. RESULTS: We found (a) differences in miRNome due to sex, SP-A genotype, and exposure; (b) miRNome of both sexes was largely downregulated by O_3_, and co-ex had fewer changed (≥2-fold) miRNAs than either group; (c) the number and direction of the expression of genes with significant changes in males and females in co-ex are almost the opposite of those in SP-A2; (d) the same pathways were found in the studied groups; and (e) O_3_ exposure attenuated sex differences with a higher number of genotype-dependent and genotype-independent miRNAs common in both sexes after O_3_ exposure. Conclusion: Qualitative differences between SP-A2 and co-ex persist 18 h post-O_3_, and O_3_ attenuates sex differences.

## 1. Introduction

Ozone (O_3_) is a reactive oxidant gas that is a major component of air pollution [1,2]. The primary effect of O_3_ occurs in the lung, causing a range of respiratory ailments [3,4,5]. The mechanism by which O_3_ mediates these effects involves the generation of reactive oxygen species (ROS), triggering oxidative stress (OxS) [6]. Several studies reported sex differences in the incidence and prognosis of pollution-induced respiratory diseases and have shown that women are at increased risk of adverse health outcomes from O_3_ and particulate matter exposure than men [7,8,9,10]. The initial defense against inhaled pathogens, allergens and air pollutants, and other harmful substances in the environment are performed by cells and molecules present in the lung. For example, the alveolar macrophage (AM), the principal effector cell for innate immunity, interacts with the innate host defense molecule, the surfactant protein A (SP-A), and together they provide the first line of defense against pathogens [11,12,13,14,15,16,17] and inhaled toxic compounds [2,18] and thus protect the lung from potential hazards by initiating a cascade of inflammatory reactions upon O_3_ exposure [2,19].

Pulmonary surfactant is a lipoprotein complex that lines the entire surface of the alveoli and prevents alveolar collapse by lowering the surface tension at the air–liquid interface of the alveoli [20]. Some of the surfactant proteins play a key role in innate immunity. Surfactant protein A (SP-A) is the major protein component of pulmonary surfactant and regulates lung innate immunity and surfactant-related functions under basal conditions [14,16,21,22,23] and in response to various insults, such as infection and OxS [24,25,26,27,28,29,30]. The human SP-A locus consists of two functional genes, *SFTPA1* and *SFTPA2*, and one pseudogene [31,32]. The functional genes encode human SP-A1 and SP-A2 proteins, respectively, and each gene has been shown to have several genetic and splice variants [31,33,34].

Previous studies have demonstrated differences between SP-A1 and SP-A2 that include both qualitative (i.e., functional, biochemical, and/or structure) [35,36,37,38,39,40,41,42,43,44,45,46,47,48] and quantitative (regulatory) changes [49,50,51,52,53,54,55,56,57,58,59]. A recent study [60] examined SP-A genotype-dependent alterations in the bronchoalveolar lavage (BAL) proteome, and explored the effects of sex, infection, and ozone-induced oxidative stress on these changes. The authors also reported changes in proteins involved in several important signal transduction pathways, including the Nuclear factor kappa-light-chain-enhancer of activated B cells (NF-kB) of the acute phase response, the nuclear factor erythroid-2-related factor 2 (Nrf2)-mediated oxidative response, and others. However, BAL proteins are derived from a variety of sources so conclusions could not be drawn about which lung cells were affected by SP-A1 and/or SP-A2. Other studies have focused on the AM and have shown that SP-A1 and SP-A2 variants differ in their ability to modulate gene expression and the proteomic expression profile of AM and the AM actin cytoskeleton [61,62,63,64]. The proteome profile of AM from the SP-A-KO mice, after treatment with exogenous SP-A1 or SP-A2, resulted in several significant changes in proteins. These included proteins involved in the OxS response pathway and actin-related cytoskeletal proteins. With regards to these processes, females were more responsive to SP-A1, whereas males were more responsive to SP-A2. The latter included proteins involved in OxS, protease balance/chaperone function, and regulation of inflammation [61]. A single-cell analysis based on actin staining revealed alveolar macrophage phenotypic subpopulations as well as sex- and age-related differences in KO mice in response to SP-A1 and SP-A2 proteins [63]. Moreover, sex differences have been observed between SP-A1 and SP-A2 and among variants in survival and lung function mechanics in response to bacterial infection [42,43]. SP-A1 compared to SP-A2 exhibits a higher efficiency in pulmonary surfactant reorganization and surfactant inhibition by serum proteins [65]. The major contributor for at least some of these differences appears to be amino acid 85 of the precursor molecule, where SP-A1 has a cysteine and SP-A2 has an arginine [31,45]. 

In the lung, miRNAs play important roles in developmental processes and maintenance of homeostasis and their dysregulation has been associated with the development and progression of various pulmonary diseases [66,67,68,69,70,71]. miRNAs can be oxidized in response to OxS, via guanine hydroxylation, altering their ability to bind target mRNA sequences [72]. In addition, miRNAs are involved in various important biological processes, such as the immune response, cell differentiation, developmental processes, and apoptosis [73,74]. The role of miRNAs in lung development was first elucidated in mice, where conditional deletion of Dicer (an important enzyme of the miRNA synthesis pathway) in lung epithelial cells resulted in impaired epithelial branching and developmental abnormalities and also led to dysregulated cell death [75]. In addition, abnormal expression of miRNAs has been correlated with the occurrence of pulmonary disorders in both children and adults [76,77,78,79,80]. Despite the known sex disparities in the incidence and severity of diseases [81,82], there are currently very few studies exploring the role of miRNAs in mediating the sex-biased disease outcomes [83]. Recent studies showed that SP-A1 and SP-A2 differentially regulate in a sex-specific manner the AM [39] and the type II cell [84] miRNome in response to O_3_ exposure. In both cases (AM and type II cell), gonadectomy had a major impact on the miRNome of males when compared to females under both control (filtered air exposure) and experimental (O_3_ exposure) conditions, indicating a likely role of sex hormones. SP-A1 by itself did not have any major effect on the AM miRNome in response to O_3_ exposure. However, in the presence of SP-A2 (i.e., in co-ex mice expressing SP-A1 and SP-A2), although there was some overlap between the two groups (SP-A2 and co-ex), some significant differences were observed at the 4 h post-O_3_ time point [39,85]), in terms of gene expression and pathways.

In the present study, we investigated whether inherent qualitative differences between SP-A2 and co-ex explain the observed differences on the AM miRNome at 4 h post-ozone exposure or whether SP-A2, in the absence of SP-A1, has a delayed response on the AM miRNome in response to O_3_. Towards this, SP-A2 (1A^0^) and co-ex (SP-A1 (6A^2^)/SP-A2 (1A^0^)) male and female mice were exposed to filtered air (FA) or O_3_ and 18 h after exposure the expression level of miRNAs, target mRNAs of the significant miRNAs, and pathways involved were studied. Mice expressing SP-A1 alone were not included in this study because our previous study found no changes in AM miRNome after a 4-h O_3_ exposure. We found significant differences in the AM miRNome in terms of genotype, sex, and exposure. The miRNome data, along with the expression levels of the validated miRNA-mRNA targets identified by IPA, as well as the IPA-identified pathways, indicated that the differences between SP-A2 and co-ex are qualitative in nature, and perhaps both gene products are needed for optimal AM regulation. Moreover, ozone appears to attenuate sex differences as more miRNAs were found to be in common between males and females in response to ozone compared to FA.

## 2. Materials and Methods

### 2.1. Animals

Twelve-week-old humanized transgenic (hTG) mice carrying a single SP-A2 (1A^0^) variant, or both SP-A1/SP-A2 (6A^2^/1A^0^, co-ex) gene variants, as well as SP-A knockout (KO) mice, were used in this study. The hTG mice were generated on the C57BL/6J SP-A (KO) background [86]. The male and female mice used in this study were raised and maintained in a pathogen-free environment, as described previously [42,43]. The females were synchronized with regards to the estrous cycle as described previously (by group housing and exposure to the bedding from male mice) [42,43]. A total of 81 mice (45 for miRNA analysis and 36 for target gene validation by qRT-PCR analysis) were used in the present study. All the procedures involving animals (protocol #44968) were approved by The Penn State Hershey Medical Center Institutional Animal Care and Use Committee (IACUC). 

### 2.2. Filtered Air (FA) and Ozone (O_3_) Exposure

The animals were exposed to FA or O_3_ (2ppm) in parallel as described previously [28,30]. This ozone dose in rodents was shown in a comparative study [87] to equate to a human dose of 0.4ppm, a level frequently encountered in urban environments. A group of 4 animals/sex/condition (FA, O_3,_ except for SP-A2 (1A^0^) and co-ex male, 3 animals/condition) for miRNA analysis and a group of 3 animals/sex/condition for target gene validation by qRT-PCR (males, females) were exposed to FA or O_3_ for 3 h, and alveolar macrophages (AMs) were isolated after 18 h of recovery as described [88].

### 2.3. RNA Preparation, Library Construction, and Sequencing 

Total RNA extraction from AM cells, library construction, and sequencing were performed as described previously [85]. The differentially expressed miRNAs between FA and O_3_-exposed males and females were identified by using the edgeR [89] and the TCC v1.14.0 R package [90] with the false discovery rate (FDR) adjusted P-value of 0.1 as a significance cutoff.

### 2.4. miRNA Data Analysis

We successfully identified 310 (SP-A2 (1A^0^)), 165 (co-ex), and 244 (KO) miRNAs (from 3 out of 4 mice, Supplementary Materials File S1). The expression levels (fold change) of miRNAs in response to FA or O_3_ from SP-A2, co-ex, and KO were analyzed and compared to identify the differentially expressed miRNAs in SP-A2, co-ex, or KO males and females. The differentially expressed miRNAs between SP-A2, co-ex, or KO males and females were determined by dividing the levels of a specific individual miRNA identified in males by the corresponding female miRNA levels and vice versa (Supplementary Materials File S1)

### 2.5. Ingenuity Pathway Analysis (IPA)

To understand the role of differentially expressed miRNAs in males and females in response to O_3_ exposure, Ingenuity Pathway Analysis (IPA, www.qiagen.com/ingenuity Qiagen, Redwood City, CA, USA) was performed as described earlier [39,85]. The miRNAs that had their levels significantly changed after comparison with ANOVA and Bonferroni correction in response to O_3_ exposure were used to identify mRNA targets.

### 2.6. Gene Expression Analysis 

Based on IPA analysis, the expression of a number of genes was validated in subsequent experiments. The expression levels of the following genes were studied by qRT-PCR as described previously [85] in male and female SP-A2, co-ex, and KO AM: AGO2, AKT1, ARG1, BCL2, CASP3, CASP8, CASP9, CCND1, CCND2, CCNE1, CDK2, CDK7, CDKN2A, CTNNB1, DDX20, E2F3, EGR2, FOXO1, GADD45A, IL6, IL10, IL2RG, JUN, MDTH, MMP9, MYC, MYD88, PPARA, PTEN, SMAD2, STAT3, TLR2, TLR3, TLR4, TNF, and TNFSF12. The RT2 qPCR Primer assays were purchased from Qiagen. The AM cell samples (3 animals/sex/treatment (FA or O_3_)) were analyzed in triplicates/animal and quantified relative to GAPDH mRNA.

### 2.7. Statistical Analysis

The statistical differences of the miRNA expression level in males and females (FA compared to O_3_ and vice versa) were evaluated by a two-tailed t-test and nonparametric Mann–Whitney test. For multiple comparison analysis, one-way analysis of variance (ANOVA) was employed followed by Bonferroni correction for multiple comparisons. Values of *p* < 0.05 were considered to be statistically significant. All the data points are means ± standard deviation, and analyses were performed using Graph-Pad Prism software version 5.0 (Graph-Pad Software, San Diego, CA, USA).

## 3. Results

### 3.1. Effect of SP-A2 (1A^0^), SP-A1/SP-A2 (6A^2^/1A^0^, co-ex), and KO on the Expression of AM miRNome 

A total of 310 (SP-A2), 165 (co-ex), and 244 (KO) miRNAs in response to filtered air (FA) or ozone (O_3_) were identified from males and females combined (listed in Supplementary Materials File S1). We observed significant differences (*p* < 0.05) in the expression of AM miRNAs between FA and O_3_ for all groups (SP-A2, co-ex, and KO) of combined male and female mice studied by the two-tailed t-test and nonparametric Mann–Whitney test (data not shown).

#### Sex Differences

**Figure 1:**
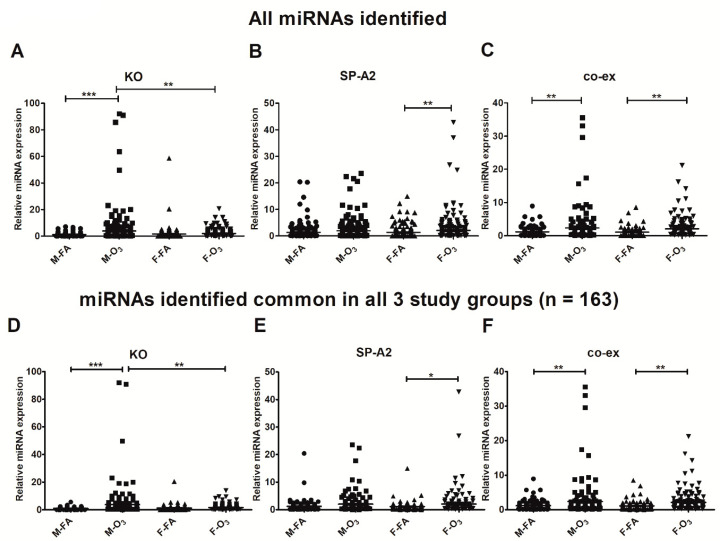
Regulation of the AM miRNome in SP-A2 (1A^0^), co-ex, and KO males (M) and females (F) after filter air (FA) and ozone (O_3_) exposure. Significant differences were determined by one-way ANOVA and subsequent Bonferroni multiple comparisons correction in KO males (**A**), and SP-A2 and co-ex females (**B**,**C**) as a function of exposure, co-ex males (**C**), and as a function of sex (KO males and females) after O_3_ exposure (**A**). Comparison analysis for the 163 miRNAs that were detectable in all of the studied groups (SP-A2, co-ex, and KO) after FA and O_3_ exposure. Significant differences are observed after Bonferroni multiple comparisons in KO males (**D**), SP-A2 and co-ex females (**E**,**F**) as a function of exposure, and co-ex males (**F**), and as a function of sex (KO males and females) after O_3_ exposure (**A**,**D**). * *p* < 0.05, ** *p* < 0.001, *** *p* < 0.0001.

One-way ANOVA and Bonferroni multiple comparison correction showed (a) no significant differences in response to FA between males and females in any of the studied groups (SP-A2, co-ex, and KO); (b) no significant differences in response to O_3_ between males and females in SP-A2 and co-ex, but in contrast, in the absence of SP-A (i.e., KO), a significant difference was observed between sexes (Figure 1A); and (c) significant differences were observed between FA and O_3_ in KO males (Figure 1A), SP-A2 females (Figure 1B), and in co-ex males and females (Figure 1C).

Next, we focused our attention on miRNAs found in common (i.e., detectable) in all the study groups (SP-A2, co-ex, and KO), after either FA or O_3_ exposure, in order to further understand the impact of SP-A gene variants (presence or absence) on miRNA expression. We identified 163 such miRNAs (Supplementary Materials File S2) and used these in comparison studies to assess the effect of SP-A2, co-ex, and KO, as well as the interaction among gene, sex, and treatment. One-way ANOVA and Bonferroni multiple comparison correction showed significant differences that were similar to the results shown in panels 1A–C, when all the identified miRNAs, and not only the ones in common, were considered. Significant differences between FA and O_3_ were observed for KO males (Figure 1D), SP-A2 females (Figure 1E), and co-ex males and females (Figure 1F). In addition, a significant difference was observed between KO males and females after O_3_ exposure (Figure 1D).

### 3.2. miRNAs, the Levels of which Changed ≥2-Fold in Response to FA, O_3_, and Sex

We studied the AM miRNAs, whose expression was altered ≥2-fold in response to FA or O_3_ from SP-A2, co-ex, and KO and compared them between males and females (Supplementary Materials File S1). To identify specific miRNA expression changes after O_3_ exposure, we compared the expression levels of miRNAs that significantly either increased (≥2-fold) or decreased (≥2-fold) in FA compared to O_3_ (FA vs. O_3_) and vice versa in males and females. The results are shown in Table 1 and Supplementary Materials File S1. A large number of miRNAs, the levels of which changed (≥2-fold either increasing or decreasing) in response to O_3_ compared to FA exposure, were observed for all three groups in both males and females (Table 1). The co-ex had the lowest number of miRNAs increased or decreased (≥2-fold) with significantly changed levels.

**Figure 2:**
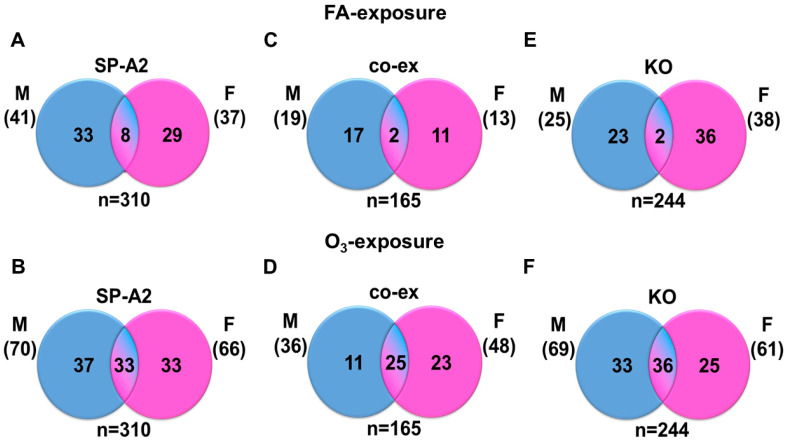
Comparison of miRNAs that are regulated (≥2-fold) by FA or O_3_ in SP-A2 (1A^0^), co-ex, and KO males and females. The changed miRNAs (≥2-fold) were used in the Venn diagrams shown. In response to FA and O_3_, for SP-A2, co-ex, and KO males and females, 310, 165, and 244 miRNAs were identified, respectively. Each Venn diagram shows the total number (n) of miRNAs for each group, the number of miRNAs specific to one or another, and the miRNAs found in common between any two groups. (**A**,**B)** show results as a function of exposure or sex for SP-A2, and similar results are shown for co-ex (**C**,**D**) and for KO (**E**,**F**). After FA **in SP-A2**, a comparison of differentially regulated miRNAs between males and females showed 41 miRNAs ≥ 2-fold in males and of these 33 were specific to males, and in females of the 37 miRNAs identified with ≥ 2-fold, 29 were specific to females. Eight miRNAs were identified to be in common between males and females after FA exposure (**A**). **In response to O_3_ exposure in SP-A2**, 70 miRNAs ≥ 2-fold were identified in males, and of these 37 were specific to males. In females, 66 miRNAs ≥ 2-fold were identified and of these 33 were specific to females. Thirty-three miRNAs were identified to be in common between males and females after O_3_ exposure (**B**). Comparable findings are shown for co-ex (**C**,**D**) and KO (**E**,**F**).

A comparison of miRNAs in males and females in response to FA or O_3_ exposure revealed the following. In response to FA, a number of the significantly (≥ 2-fold) changed miRNAs, shown in Table 1, were found to be in common in males and females, indicating that the expression of these miRNAs is independent of sex. These included eight miRNAs for SP-A2, two miRNAs for co-ex, and two miRNAs for KO, leaving the expression of the majority of the changed miRNAs under the FA condition to be present only in either males or females. However, in response to O_3_, a significantly larger number of miRNAs were found to be in common between males and females in all three groups. SP-A2 had 33 in common, co-ex had 25, and 36 for KO (Figure 2, Supplementary Materials File S1). 

### 3.3. Shared miRNAs among the Three Studied Groups in Response to FA or O_3_ in Males and Females

**Figure 3:**
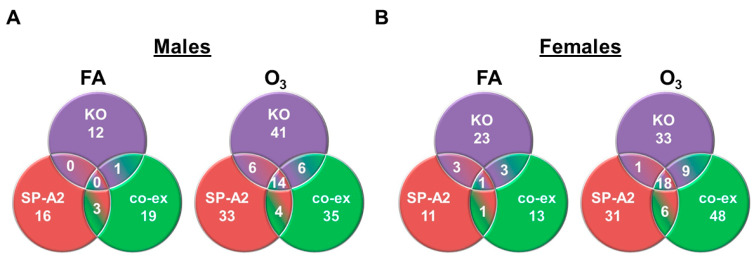
The Venn diagrams show miRNAs present in all 3 study groups that changed ≥2-fold in response to FA or O_3_. miRNAs (*n* = 163) were identified to be in common in all 3 study groups. Out of 163 miRNAs, in response to FA, no miRNAs (≥2-fold) were found to be in common in males in the three groups, but one miRNA (≥2-fold) was found to be in common in the three groups in females (**A**,**B**). In response to O_3_ exposure, 14 and 18 miRNAs (≥2-fold) were in common in the three study groups in males and females, respectively (**A**,**B**). The miRNAs found to be changed (≥2-fold) in all three groups are likely independent of SP-A regulation.

For this analysis, we used miRNAs (*n* = 163) found to be present/detectable (regardless of the level of expression) in all three groups. The Venn diagrams in Figure 3 show that in response to FA, there are either no miRNAs (males) or only one miRNA (females) in common among the three groups. However, in response to O_3_, there is a significantly higher number of miRNAs found in common in males (*n* = 14) and females (*n* = 18) among the three groups (Figure 3, Supplementary Materials File S2). Collectively, the data in Figure 2 and Figure 3 indicate that in response to O_3_, although gene/genotype- and sex-specific differences remain in terms of the AM miRNome compared to FA, more miRNAs are found to be in common among the three groups in males and females.

### 3.4. Ingenuity Pathway Analysis (IPA) Pathways

IPA was performed to identify target genes of the significantly changed miRNAs and pathways involved in SP-A2, co-ex, and KO males and females under the studied conditions. The results of the IPA analysis were subjected to one-way ANOVA before and after the Bonferroni correction for each study set (i.e., SP-A2 females in FA vs. O_3_) yielded identical signaling networks and pathways because the miRNA data input in the IPA was same. The target genes of the differentially expressed miRNAs identified by IPA are involved in anti-apoptosis, cell cycle, cellular growth and proliferation, as well as proinflammatory responses. The miRNA target genes included AGO2, AKT1, ARG1, BCL2, CASP3, CASP8, CASP9, CCND1, CCND2, CCNE1, CDK2, CDK7, CDKN2A, CTNNB1, DDX20, E2F3, EGR2, FOXO1, GADD45A, IL6, IL10, IL2RG, JUN, MDTH, MMP9, MYC, MYD88, PPARA, PTEN, SMAD2, STAT3, TLR2, TLR3, TLR4, TNF, and TNFSF12. The miRNAs that significantly changed in response to O_3_ exposure and their targets in SP-A2, co-ex, and KO males and females are listed in Table 2. 

### 3.5. Validation of miRNA Target Genes

**Figure 4:**
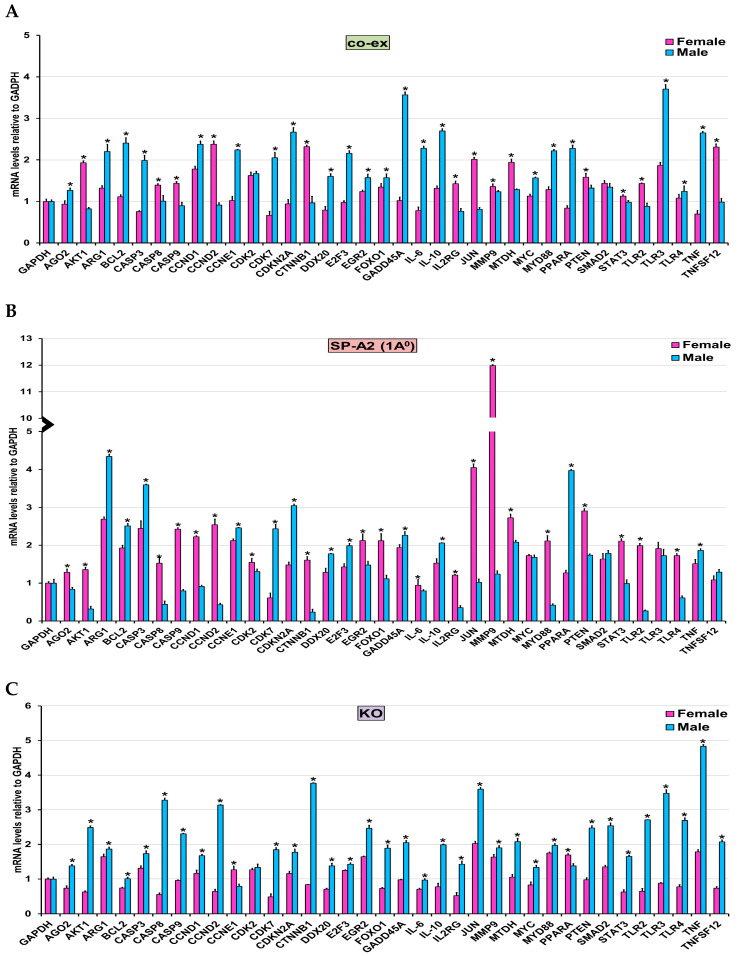
Effect of O_3_ exposure on mRNA targets of co-ex, SP-A2 (1A^0^), and KO. (**A**–**C)** show the gene expression levels for co-ex, SP-A2, and KO, respectively, in males and females. **In co-ex** (**A**), the expression of the levels of AGO2, ARG1, BCL2, CASP3, CCND1, CCNE1, CDK7, CDKN2A, DDX20, E2F3, EGR2, FOXO1, GADD45A, IL-6, IL-10, MYC, MYD88, PPARA, TLR3, TLR4, and TNF were significantly upregulated in males compared to females. The levels of AKT1, CASP8, CASP9, CCND2, CTNNB1, IL2RG, JUN, MMP9, MTDH, PTEN, STAT3, TLR2, and TNFSF12 were upregulated in females compared to males. The levels of CDK2 and SMAD2 remained similar in both sexes. Levels of expression of the studied genes in SP-A2 (1A^0^) and KO are shown in (**B**,**C**), respectively. Blue and pink bars show the expression levels of specific genes in males and females, respectively. The expression levels were normalized to GAPDH and significant differences (*p* < 0.05) between sexes are noted by an asterisk (*).

The expression of miRNA target genes identified by IPA was next validated by qRT-PCR analysis on AM cell samples isolated from SP-A2, co-ex, and KO males and females after FA or O_3_ exposure (Figure 4). In response to O_3_, the expression levels of 12 genes: ARG1, BCL2, CASP3, CCNE1, CDK7, CDKN2A, DDX20, E2F3, GADD45A, IL-10, PPARA, and TNF, significantly increased in co-ex and SP-A2 males compared to females (Figure 4A,B) whereas the expression levels of another 12 genes: AKT1, CASP8, CASP9, CCND2, CTNNB1, IL2RG, JUN, MMP9, MTDH, PTEN, STAT3, and TLR2, significantly increased in co-ex and SP-A2 females compared to males (Figure 4A,B). The expression levels of the other validated genes were discordantly either increased or decreased between SP-A2 and co-ex males and females. The expression levels of MYC, SMAD2, TLR3, and TNFSF12 in SP-A2 (Figure 4B), and CDK2 and SMAD2 in co-ex (Figure 4A) remained similar between sexes. In contrast, in SP-A-KO, in response to O_3_, the expression levels of nearly all of the genes studied significantly increased in males compared to females (Figure 4C), with the exception of CDK2, which did not change between sexes, and the CCNE1, and PPARA, which significantly increased in females in response to O_3_ compared to males (Figure 4C).

In summary, in the absence of SP-A, in KO, the overwhelming majority of the validated target genes (*n* = 33) showed increased expression in males compared to females. Two showed a significant increase in females compared to males and one gene (CDK2) showed no sex differences. In the presence of the SP-A2, a larger number of genes in females (*n* = 20) exhibited increased expression compared to males (*n* = 12). Four genes (MYC, SMAD2, TLR3, and TNFSF12) did not show sex differences in their expression. In the presence of both gene products (co-ex), similarly to SP-A2, there was a large number of genes that exhibited increased expression. Two genes (CDK2 and SMAD2) did not show sex differences in their expression. However, in co-ex, the larger number of genes that exhibited increased expression was in males (*n* = 21) than in females (*n* = 13). The number and direction of expression (increase or decrease) of genes that showed a significant change in co-ex males or females is almost the opposite of what is observed in the presence of SP-A2, where the larger number of genes exhibiting increased expression was in females. This may indicate an interactive role of SP-A1 and SP-A2 in the observed sex differences. The presence of SP-A1 (in addition to SP-A2) may bring about a downregulation of miRNA-target genes in females, and a relative upregulation in males as depicted in co-ex vs. SP-A2 alone. In the absence of SP-A, as shown in the KO, there is an overwhelming upregulation in males. Thus, both gene products are required for perhaps a more balanced gene expression in males and females. This is consistent with the lower number of miRNAs with changed levels observed in co-ex than either SP-A2 or KO in response to O_3_ (Table 1).

### 3.6. In Response to O_3_ Exposure

#### 3.6.1. SP-A Genotype-Independent miRNAs (i.e., Found in Common among the Three Groups (SP-A2, co-ex, KO))

Of the miRNAs (*n* = 163) found to be in common in all three groups (SP-A2, co-ex, and KO) and changing in response to O_3_ exposure, 14 and 18 miRNAs (≥2-fold) were present in males and females, respectively, in all 3 groups (Figure 3 and Supplementary Materials File S2), indicating that these are SP-A genotype independent. Out of these, 13 miRNAs were found to be in common in both males and females. The IPA analysis of these (*n* = 14 in males; *n* = 18 in females) miRNAs identified target genes predicted (TargetScan) to be involved in proinflammatory (TNF, TNFSF12, TLR2, TLR3, and TLR4), cell cycle, growth, and proliferation (CCND1, CCND2, CCNE1, CDK7, E2F3, JUN, PPARA, and PTEN). The expression levels of these target genes were significantly altered in response to O_3_ exposure in males and females, as shown in Figure 4. 

Although a substantial number of the miRNAs used in IPA were found to be in common (*n* = 13) in males and females after O_3_ exposure, their target gene expression differed between males and females in all three groups, except for TLR3 and TNFSF12, which did not change between sexes in SP-A2. Thus, other mechanisms may contribute directly or indirectly to the regulation of the target genes of these SP-A genotype-independent miRNAs.

#### 3.6.2. SP-A Genotype-Dependent miRNAs (i.e., Not Found in Common among the Three Groups)

IPA of miRNAs that are not common (*n* = 149 and 145 for males and females, respectively) in all three groups (SP-A2, co-ex, and KO) (Supplementary Materials File S2) in response to O_3_ exposure showed that the targets of these miRNAs are associated with the same pathways as noted above for the miRNAs found to be in common among the three groups. These include proinflammatory (IL6, MTDH, TLR2, TLR3, TNF, and TNFSF12), and cell cycle, growth, and proliferation (CCND1, CCNE1, CTNNB1, CDK7, E2F3, FOXO1, and PPARA). 

In summary, it is evident that the group of miRNAs found to be in common in all three study groups and the group of miRNAs not found to be in common in the three study groups, although different, some of these, target the same genes. The pathways identified by IPA based on the miRNome in response to O_3_ exposure include proinflammatory and cell cycle, growth, and proliferation pathways. We speculate that AMs initiate these pathways as recovery mechanisms to alleviate the impact of O_3_ exposure regardless of SP-A genotype or lack of SP-A, but the degree of success may in part depend on the combination of miRNAs that regulate the genes involved. This putative combination of miRNAs may consist, among others, of miRNAs specific to a given SP-A genotype, miRNAs specific to males or females, and miRNAs specific to the interaction of SP-A genotype/sex/O_3_ exposure.

**Figure 5:**
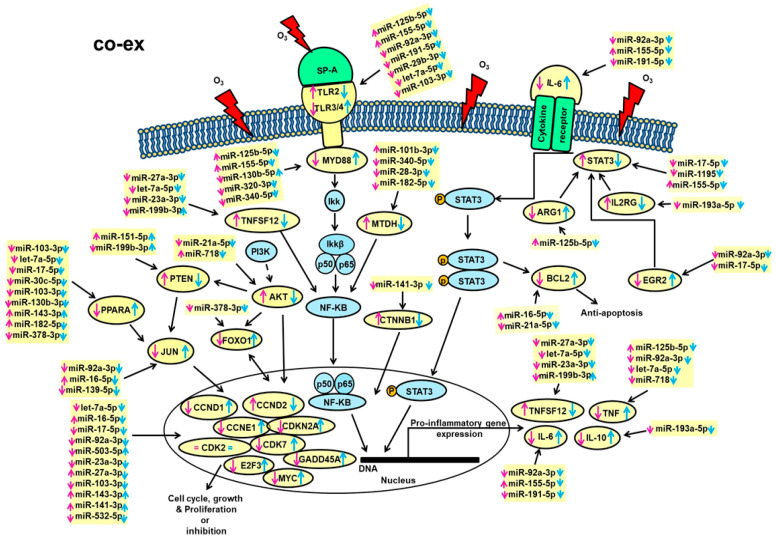
Schematic representation of the identified miRNAs in co-ex AM and their targets in response to O_3_. These include pathways of cell cycle, and cellular growth and proliferation, as well as pathways of the proinflammatory response and anti-apoptosis, in co-ex males and females. The miRNAs and their gene targets studied in the present study are highlighted with yellow. Up (↑) and down (↓) arrows in blue and pink color indicate an increase and decrease, respectively, in males or females.

The collective information indicates that O_3_ exposure has a significant impact on the expression of miRNAs and their target genes in an SP-A genotype-specific and sex-specific manner. A pictorial integration and summary of all molecules studied here is shown in Figure 5 for the co-ex. These include the significantly changed miRNAs, their validated targets, and the signaling pathways identified by IPA. Supplementary Materials Figure S1A,B depict a similar summary for SP-A2 and KO, respectively, as shown in Figure 5 for co-ex. 

## 4. Discussion

Four hours after a 3 h O_3_ exposure, both sex- and SP-A gene-specific differences were observed in the AM miRNome, with SP-A2 males exhibiting significant differences [39]. No significant differences were observed in AMs from mice expressing SP-A1. AMs from mice that expressed both gene products (co-ex) [85] when compared to SP-A2 male mice after O_3_ exposure [39] exhibited both similarities and differences in the miRNA-targeted genes and pathways [85]. In the present study, we wished to investigate the effect of O_3_ exposure at a later time point to determine whether the AM miRNome from SP-A2 mice exhibits a delayed response compared to co-ex or whether it remains qualitatively different from that in co-ex. Towards this, human transgenic (hTG) mice, expressing SP-A2 (1A^0^), or both gene products (co-ex), and SP-A-KO were exposed to filtered air (FA) and O_3_ and AM miRNA levels were identified at 18 h after a 3 h O_3_ exposure. The target genes of the significant miRNAs were validated and studied by IPA to identify signaling pathways. The observations made include (i) significant differences in AM miRNome of SP-A2, co-ex, and KO in terms of sex and exposure; (ii) the AM miRNome was largely downregulated significantly in response to O_3_ compared to the control (FA) in both males and females in all studied groups; (iii) the expression of the overwhelming majority of miRNA targets in KO males was increased compared to females. In SP-A2, about 56% of the targets showed an upregulation in females compared to males, whereas in co-ex, the opposite was observed, with 58% being upregulated in males; (iv) miRNA-mRNA targets of all three study groups were involved in proinflammatory response, anti-apoptosis, cell cycle, cellular growth, and proliferation pathways. These data indicate that although the overwhelming majority of miRNAs are downregulated in response to O_3_ and similar pathways are observed for the three study groups, the expression of the miRNA-mRNA targets differs as a function of SP-A genotype and sex. This indicates that mechanisms other than those mediated by miRNA play a role. Moreover, the presence of SP-A1 as shown in co-ex appears to play a significant role in the regulation of miRNA targets in a sex-specific manner.

### 4.1. Anti-Apoptosis, Cell Cycle, Growth, and Proliferation

The mRNA levels of anti-apoptotic protein BCL2 were significantly increased in both SP-A2 and co-ex males but decreased in females at 18 h post-O_3_. This is consistent with observations made at 4 h post-O_3_ exposure [39,85]. Several studies showed that the expression of BCL2 was significantly increased in response to various environmental insults [91,92,93]. The expression of miR-16-5p and miR-21a-5p, which target BCL2 [94,95,96,97], was significantly decreased in the present study in both SP-A2 and co-ex except in co-ex females where miR-16-5p increased. Although O_3_ differentially affects BCL2 expression in males and females, there appears to be a disconnect between miRNA expression and target gene expression, indicating that miRNAs not studied here or other mechanisms contribute directly or indirectly to the BCL2 regulation after O_3_ exposure. 

O_3_ exposure differentially affected the expression of molecules involved in cell cycle and growth and proliferation in the studied groups. A number of miRNAs whose expression was for the most part downregulated after O_3_ exposure were predicted to target genes involved in cell cycle and growth and proliferation pathways, such as CCND1, CCND2, CCNE1, CDK2, CDK7, CDKN2A, E2F3, GADD45A, and MYC (Table 2). For example, miR-16-5p and miR-17-5p, which are predicted to bind CCND1, CCND2, CCNE1, CDK7, E2F3, and MYC mRNAs, have been shown in several studies to regulate these genes [98,99,100,101]. The mRNA levels of CCNE1, CDK7, CDKN2A, E2F3, and GADD45A were increased in males, which is consistent with the downregulation of miRNAs regulating their expression. In females, on the other hand, there was a decreased expression of these genes in spite of the miRNA downregulation. The opposite was observed for other genes in this pathway. The mitogen-activated protein kinase (MAPK) pathway has the potential to regulate genes involved in cell cycle, growth, and proliferation, as well as proinflammatory and anti-apoptosis [102,103,104]. In the present study, we found the expression of several genes involved in the MAPK pathway to be altered after O_3_ exposure. For example, an increase of FOXO1 was observed in both co-ex and SP-A2 males, and the miRNA that targets FOXO1 and miR-378-3p was significantly downregulated in both co-ex and SP-A2 (Figure 5 and Supplementary Materials Figure S1B). FOXOs are transcription factors involved in the homeostasis of ROS and can function as a negative feedback loop to control cellular reactive oxygen species [105]. FOXO1 regulates the expression of antioxidant genes, such as CAT and SOD2, both of which are known to neutralize free radicals generated by ROS. We have previously shown that at 18 h post-O_3_ exposure, the level of SOD2 mRNA was decreased in SP-A2 males, whereas, in KO, both SOD2 and CAT were significantly increased, indicating that SP-A2 may play a role in the homeostasis of ROS [39]. In fact, our recent studies indicate that SP-A2 contributes/regulates the NAD(H) redox status in a sex-dependent manner [106]. Thus, the observed FOXO1 upregulation may be a mechanism that alleviates the ROS impact on AM cells of male SP-A2 and co-ex compared to females. 

### 4.2. Proinflammatory Responses

The pro- and anti-inflammatory cytokine IL-6 is regulated by miR-191-5p, miR-155-5p, and miR-92-3p [39,107], the expression of which was variably changed in the study groups. Of these, the up- and downregulation of miR-155-5p was consistent in the present study with the down- and upregulation of IL-6 in the various groups. However, the expression level of the other two miRNAs predicted to bind IL-6 was not consistent with the IL-6 expression levels. miR-155-5p is a multi-functional miRNA that regulates inflammatory signaling pathways [108], and is shown to negatively regulate the IL-6-triggered proinflammatory pathway by preventing Janus kinase 2 (JAK2)/signal transducer and activator of transcription 3 (STAT3) activation [107,109]. IL-6 is secreted by immune cells and lung endothelial and epithelial cells in response to environmental insults [110,111], and via its pleiotropic effects modulates pathogenesis, progression, and severity of various chronic lung diseases [112,113,114]. The level of IL-6 significantly decreased in SP-A2 males compared to females, whereas the opposite was observed in co-ex males and females at 18 h post-O_3_ exposure. However, at 4 h post-O_3_, an increase was observed in males of SP-A2 or co-ex [39,85]. This variable IL-6 regulation in the presence of the SP-A2 single gene product and in co-ex indicates an interplay of both gene products in the IL-6 regulation. 

Moreover, MMP9, which has been implicated in the pathogenesis of several lung diseases and injury [115,116,117,118], has been shown to increase the expression of IL-6 in response to O_3_ [119]. The expression of MMP9, on the other hand, is enhanced in AM cells and other cells in the lung by various stimuli, and specifically by ozone [120,121,122]. In the present study, the expression of let-7a-5p and miR-320-3p, predicted to bind MMP9, was downregulated in both SP-A2 and co-ex, and MMP9 expression was increased in both SP-A2 and co-ex females compared to males, but in KO, its expression was instead increased in males vs. females. The increase in MMP9 expression may upregulate the expression of IL-6 in SP-A2 (but not in co-ex) females as shown in a previous study with wild-type mice [119].

Intracellular signaling mediated by STAT3 has been implicated in lung inflammation and in the pathogenesis of various lung diseases [123,124,125,126,127,128]. In response to O_3_, STAT3 gets phosphorylated and this results in the activation of genes involved in inflammation and injury [129]. miR-17-5p, miR-1195, and miR-155-5p are predicted to bind and regulate STAT3. The expression of these miRNAs was variably changed in the study groups in response to O_3_. Of these, the expression of miR-1195 is consistent with the observation made. Its decreased expression in SP-A2 and co-ex (males and females) is associated with increased expression of STAT3 in SP-A2 and co-ex females compared to males. Although some of the miRNAs were downregulated in males after 18 h post-O_3_, which should have resulted in an increase in the expression of STAT3, the opposite was observed, indicating that either the regulation of STAT3 by these (and perhaps other) miRNAs is dysfunctional or mechanisms other than miRNAs regulate its expression in males at 18 h post-O_3_. This differs from a previous observation where males showed a significant increase in STAT3 levels in response to 4 h post-O_3_ [39,85], indicating that in the presence of the single SP-A2 or both genes, STAT3 expression is differentially regulated in males and females at different time points, as a function of SP-A genotype. Moreover, a number of target genes involved in the regulation of the proinflammatory cytokines via STAT3 were significantly and variably altered in response to O_3_. However, the role of these miRNA target genes in regulating the pro- or anti-inflammatory response to environmental pollutants in males and females remains unexplored as a function of SP-A genotype. 

In general, the up- and downregulation of several of the genes studied in the various groups did not fully correlate with changes in the miRNAs that targeted these genes, as most miRNAs exhibited a downregulation. The varied gene expression was largely sex and SP-A genotype specific. Sex-specific and SP-A genotype-dependent differences in survival after infection [42] and O_3_ [25,27] have been shown in animal studies and sex hormones were shown to play a role [130]. These together point to underlying complexities of sex-mediated mechanisms in response to SP-A genotype that cannot be addressed by the miRNome alone, although miRNAs may partially contribute to mechanisms in response to various insults. 

## 5. Overall Comments 

SP-A-KO mice exhibited significantly poor survival after infection compared to wild-type mice [27] or compared to humanized transgenic mice where each expresses a different SP-A variant. Rescue with SP-A significantly improved survival [42], and the proteomic profile of the rescued KO AM resembled that of the wild type [131]. Observations, however, of the KO AM proteome [29,132], miRNome [39,85], or gene expression [133] are somewhat perplexing or paradoxical. These, among others, indicated that unchallenged AMs in the absence of SP-A may be in a state of OxS [29] as suggested by previous studies, where, after infection, the KO AM proteome was reminiscent of the uninfected wild-type proteome [132]. In the latter, proteins with pathogen defense functions were increased in the KO, indicating perhaps an attempt for the KO to overcome its host defense deficits. Similarly, in the present study, the KO AM miRNome shared similarities with SP-A2 and co-ex. However, in spite of various attempts of the KO AM to respond to insults in ways that may resemble those of the SP-A-expressing mice, they cannot overcome their host defense deficits as shown by their poor survival following infection [26,27,42]. A recent paper using a Toponomic imaging system provided some insight into “pattern of expression” or combinational molecular phenotypes (CMPs) within individual cells [134]. In an SP-A1 rescue experiment, the patterns of expression or CMPs overlapped between KO and SP-A1-rescued KO AM, and the rescued AMs exhibited more diversity in the form of CMPs than the KO. This diversity/heterogeneity and the overlap of CMPs may in part explain the AM functional differences [25,26,27], where the presence of SP-A lead to a better outcome as shown with the ultimate readout survival [26,27,42].

Although, SP-A1 alone in the original study [39] did not show any significant effect on the AM miRNome at 4 h after O_3_ exposure, together with SP-A2 in co-ex, and regulated genes involved in the cell cycle, anti-apoptosis, and growth and proliferation pathways as early as 4 h post-O_3_ [85]. This is in contrast to SP-A2 males, where the cell cycle pathway was not detected at the 4 h time point but instead, the ROS homeostasis pathway was identified as playing a role [39]. One may postulate that in the absence of SP-A1, SP-A2 alone at the 4 h post-O_3_ time point affects ROS-related mechanisms and one of these mechanisms may be via its ability to regulate the NAD(H) redox status [106]. However, at a later time point (18 h), SP-A2 shifts to recovery mechanisms by perhaps activating the cell cycle, growth, and proliferation pathway (present study). However, even though the general pathways identified here for the three study groups (SP-A2, co-ex, and KO) were the same at 18 h, the processes involved in the regulation of the miRNome and the miRNA-mRNA targets differed significantly among the groups. The co-ex compared to SP-A2 had a lower total number of miRNAs either increased or decreased in response to O_3_ exposure. It was 49% (males) and 59% (females), approximately half of those in SP-A2. The expression of most of the target genes increased in SP-A2 females compared to males, but the opposite was true for the co-ex. Collectively, these indicate that a) qualitative differences exist between AM from SP-A2 and co-ex up to at least 18 h after O_3_ exposure, b) the presence of both genes may be necessary for optimal AM functioning, and c) a regulatory interplay between SP-A1 and SP-A2 may exist, even though the SP-A1 by itself did not show any significant differences at 4 h post-O_3_ exposure in either males or females [39]. In humans with no known pulmonary disease, the ratio of SP-A1 to total SP-A in bronchoalveolar lavage (BAL) varies [54]. However, this ratio increases significantly in patients with cystic fibrosis, asthma, and positive bacterial culture [54]. Thus, a putative relative imbalance of the SP-A gene expression products in BAL may under certain conditions contribute to lung disease. This postulate is in part supported by animal studies, where animals with single-gene products survive equally well under unprovoked conditions, but in response, for example, to infection, their survival varies significantly [42]. Although the data of this study are largely in line with our previous observations, the current study has a few limitations: (a) the validation analysis was performed only for genes that are targeted by significantly changed miRNAs in co-ex, SP-A2, and KO males and females, and we did not look at the protein levels of the targeted mRNAs; and (b) we did not study the molecular mechanisms of the identified pathways. However, the result of this study advances our knowledge of the differential impact of SP-A genotype and sex on the AM miRNome in response to O_3_ exposure. 

## 6. Conclusions

(a) In response to O_3_ qualitative differences in the AM miRNome, target genes and signaling pathways were observed as a function of SP-A genotype and sex. (b) Fewer miRNAs were increased or decreased (≥2-fold) in co-ex than either group (SP-A2, KO). (c) O_3_ exposure attenuated sex differences as shown: (i) more miRNAs were found to be in common between males and females in each studied group compared to FA exposure, with co-ex having the lower number than either group (SP-A2 or KO); and (ii) more miRNAs were found to be in common in males or females among the three studied groups (i.e., regardless of SP-A genotype) compared to FA exposure. Together, these point to underlying complexities and an interplay of O_3_ effects, sex, and SP-A genotype. 

## Figures and Tables

**Table 1:** The total numbers of miRNAs identified from SP-A2 (1A^0^), co-ex, and KO males and females with ≥2-fold change after FA and O_3_ exposure are shown.

Gene Variant and Number of miRNAs Identified	Male		Female	
	FA vs. O_3_		FA vs. O_3_	
	≥2-fold (Increase)	≥2-fold (Decrease)	≥2-fold (Increase)	≥2-fold (Decrease)
SP-A2 (1A^0^) (*n* = 310)	41 *	70 *	37 *	66 *
co-ex (*n* = 165)	19 *	36 *	13 *	48 *
KO (*n* = 244)	25 *	69 *	38 *	61 *

The number (*n* = ) of miRNAs analyzed is shown in parentheses in the first column. * Number of miRNAs that significantly changed ≥2-fold either increasing or decreasing in FA (Filtered air) compared to O_3_ (FA/O_3_) in males and females.

**Table 2:** Expression levels of SP-A2 (1A^0^), co-ex, and KO AM miRNAs (males and females) in response to O_3_ exposure that significantly changed, and their mRNA targets were identified by IPA analysis.

miRNA ID	SP-A2 (1A^0^)		co-ex (SP-A1 (6A^2^)/SP-A2 (1A^0^)		KO		Target Molecule
	Fold Change in Males	Fold Change in Females	Fold Change in Males	Fold Change in Females	Fold Change in Males	Fold Change in Females	
let-7a-5p	1.395	0.942	0.912	1.356	0.498	1.020	AGO2, CCND1, CCND2, CCNE1, CDKN2A, CDK7, E2F3, MMP9, PPARA, TLR4, TNF, TNFSF12
miR-16-5p	1.621	0.787	1.045	2.301 †	0.620	0.929	CCND1, CCND2, CCNE1, CDK7, TNFSF12, E2F3, BCL2, JUN
miR-17-5p	1.994 †	0.734	0.960	0.953	0.212 †	0.199 †	CCND1, CCND2, CCNE1, CDK7, STAT3, EGR2, E2F3, MYC, PPARA, TNFSF12
miR-21a-5p	0.649	0.553	1.338	0.563	0.966	0.994	BCL2, AKT
miR-23a-3p	1.111	0.842	0.808	0.895	0.782	1.225	E2F3, TNFSF12
miR-25-5p	0.692	1.692	0.557	0.807	1.219	0.235 †	SMAD2
miR-27a-3p	1.253	1.085	0.960	2.045 †	1.163	1.206	E2F3, TNFSF12
miR-28-3p	1.216	0.587	0.203 †	0.826	0.177 †	0.843	MTDH
miR-29b-3p	0.546	1.402	1.540	1.258	2.539 †	0.707	AGO2, TLR3
miR-30c-5p	1.293	0.870	0.976	0.987	1.617	0.864	AGO2, DDX20, PPARA
miR-101b-3p	0.456 †	1.146	1.153	2.339 †	1.001	1.147	MTDH
miR-103-3p	1.027	0.869	1.219	1.108	0.680	1.005	E2F3, PPARA, AGO2, TLR4
miR-125b-5p	1.556	6.921 †	1.643	5.025 †	3.585 †	2.030 †	TLR2, TNF, ARG1, MYD88
miR-130b-3p	4.656 †	3.584 †	0.128 †	0.505	1.207	0.554	PPARA
miR-130b-5p	2.613 †	1.204	2.263 †	0.420 †	0.582	0.236 †	MYD88
miR-139-5p	5.637 †	2.121 †	0.961	1.422	3.378 †	1.527	AGO2, JUN
miR-141-3p	0.888	1.362	2.015 †	6.530 †	6.623 †	5.760 †	CTNNB1, GADD45A
miR-143-3p	3.613 †	4.148 †	4.202 †	16.191 †	9.130 †	5.773 †	E2F3, PPARA
miR-151-5p	1.345	2.847 †	35.602 †	7.713 †	2.508 †	1.670	PTEN, AGO2
miR-155-5p	6.792 †	0.192 †	1.077	2.088 †	0.421 †	2.479 †	IL-6, TLR2, MYD88, STAT3
miR-181a-5p	0.788	0.970	0.578	0.585	0.875	1.040	SMAD2
miR-182-5p	1.622	1.216	0.375 †	3.279 †	1.984	1.235	PPARA, MTDH
miR-191-5p	1.311	1.093	1.029	0.960	0.707	1.017	IL-6, TLR3
miR-193a-5p	0.677	0.842	0.758	0.701	1.277	0.796	IL-10, IL2RG
miR-199b-3p	5.455 †	2.659 †	1.829	0.543	7.971 †	3.341 †	PTEN, TNFSF12
miR-320-3p	0.802	1.498	0.994	0.942	1.072	0.649	MYD88
miR-320b	1.114	0.324 †	0.700	0.876	0.333 †	0.773	MMP9, SMAD2
miR-340-5p	0.843	0.748	1.607	0.646	2.031 †	0.438 †	MTDH, MYD88
miR-378-3p	1.246	0.631	0.764	1.004	0.778	0.808	PPARA, FOXO1, CASP9
miR-455-3p	3.142 †	5.087 †	2.554 †	3.777 †	4.915 †	3.509 †	TNFSF12
miR-503-5p	0.934	0.984	2.333 †	0.919	0.498	1.468	CDK2
miR-532-5p	1.235	0.632	0.424 †	0.922	0.566	0.795	MYC
miR-92a-3p	1.338	2.008 †	0.903	0.888	1.131	0.380 †	CCND1, CCNE1, CDK7, IL-6, TLR2, TLR3, EGR2, JUN, E2F3, TNF, SMAD2
miR-718	1.053	0.932	0.819	2.754 †	1.214	1.187	TNF, AKT
miR-1195	1.392	0.431 †	0.375 †	0.699	0.655	0.767	STAT3

† indicates miRNAs that had expression value with either ≥2-fold increase (values ≥ 2) or ≥2-fold decrease (values ≤ 0.5), and are highlighted in yellow (*p* < 0.05).

## Data Availability

The datasets generated for this study are included in the manuscript and the Supplementary Files, and has been deposited in the Gene Expression Omnibus repository GSE158401 (https://www.ncbi.nlm.nih.gov/geo/query/acc.cgi?acc=GSE158401).

## Supplementary Materials

The following are available online at https://www.mdpi.com/2076-3921/9/12/1190/s1, Supplementary File S1: Identification of miRNAs from AMs of SP-A2 (1A^0^), co-ex, and KO males and females in response to Filtered air and Ozone after 18 h recovery. File includes all raw data for each sample, group means, *p* values, fold changes (≥2-fold, highlighted in green (FA), and red (O_3_) exposure). Supplementary File S2: miRNAs (*n* = 163) identified to be in common among SP-A2 (1A^0^), co-ex, and KO after FA and O_3_ exposure. Supplementary Figure S1. Schematic representation of the identified miRNAs in SP-A2 (1A^0^), and KO AM and their targets in response to O_3_. These include pathways of cell cycle, and cellular growth and proliferation as well as, pathways of pro-inflammatory response and anti-apoptosis, in SP-A2 (1A^0^) (A), and KO (B) males and females. The miRNAs and their gene targets studied in the present study are highlighted with yellow. Up (↑) and down (↓) arrows in blue and pink color indicate an increase and decrease, respectively, in males or females.

## Abbreviations

AGO2: Argonaute 2; AKT1: AKT Serine/Threonine Kinase 1; ARG1: Arginase 1; AM: Alveolar macrophages; ANOVA: Analysis of variance; BAL: bronchoalveolar lavage; BCL2: B-cell lymphoma 2; CASP3: Caspase 3; CASP8: Caspase 8; CASP9: Caspase 9; CCND1: Cyclin D1; CCND2: Cyclin D2; CCNE1: Cyclin E1; CDK2: Cyclin-dependent kinase 2; CDK7: Cyclin-dependent kinase 7; CDKN2A: Cyclin dependent kinase inhibitor 2; CTNNB1: Catenin alpha 1; DDX20: Dead-box helicase 20; E2F3: E2F transcription factor3; EGR2: Early growth response 2; FA: Filtered air; FOXO1: Forkhead box O1; GADD45A: Growth arrest and DNA damage inducible alpha; GAPDH: Glyceraldehyde 3-phosphate dehydrogenase; hTG: Humanized transgenic; IL6: Interleukin 6; IL10: Interleukin 10; IL2RG: Interleukin 2 receptor subunit gamma; IPA: Ingenuity Pathway Analysis; JUN: Jun proto-oncogene; KO: knock-out; MDTH: Metadherin; miRNAs: microRNAs; MMP9: Matrix metalloproteinase 9; MYC: MYC proto-oncogene; MYD88: Myeloid differentiation primary response 88; NF-kB: Nuclear factor kappa-light-chain-enhancer of activated B cells; O3: ozone; OxS: oxidative stress; PPARA: Peroxisome proliferator activated receptor alpha; PTEN: Phosphatase and tensin homolog; ROS: reactive oxygen species; SFTPA1: gene encoding SP-A1; SFTPA2: gene encoding SP-A2; SMAD2: SMAD family member 2; SP-A: surfactant protein A; STAT3: Signal transducer and activator of transcription 2; TLR2: Toll-like receptor 2; TLR3: Toll-like receptor 3; TLR4: Toll-like receptor 4; TNF: Tumor necrosis factor; TNFSF12: TNF super family member 12.
