# Supplementary figures and images for: Differential Sex-Dependent Regulation of the Alveolar Macrophage miRNome of SP-A2 and co-ex (SP-A1/SP-A2) and Sex Differences Attenuation after 18 h of Ozone Exposure
Source: Antioxidants (Basel). 2020 Nov 27;9(12):1190. doi: 10.3390/antiox9121190 (PMC7768498; doi:10.3390/antiox9121190)

Supplementary Figure 1  
SP-A2 (1A<sup>0</sup>)

A

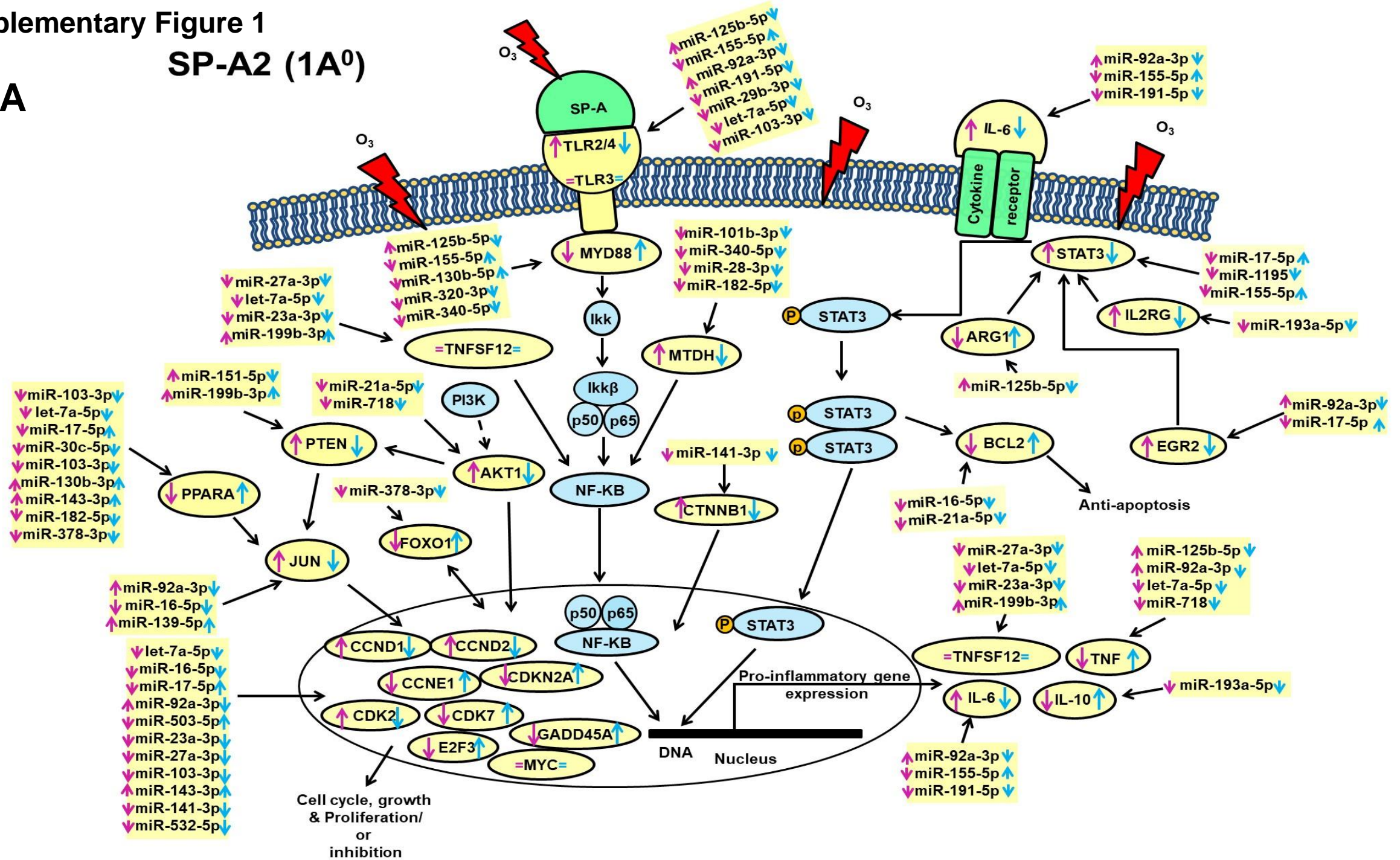

Supplementary Figure 1

B

KO

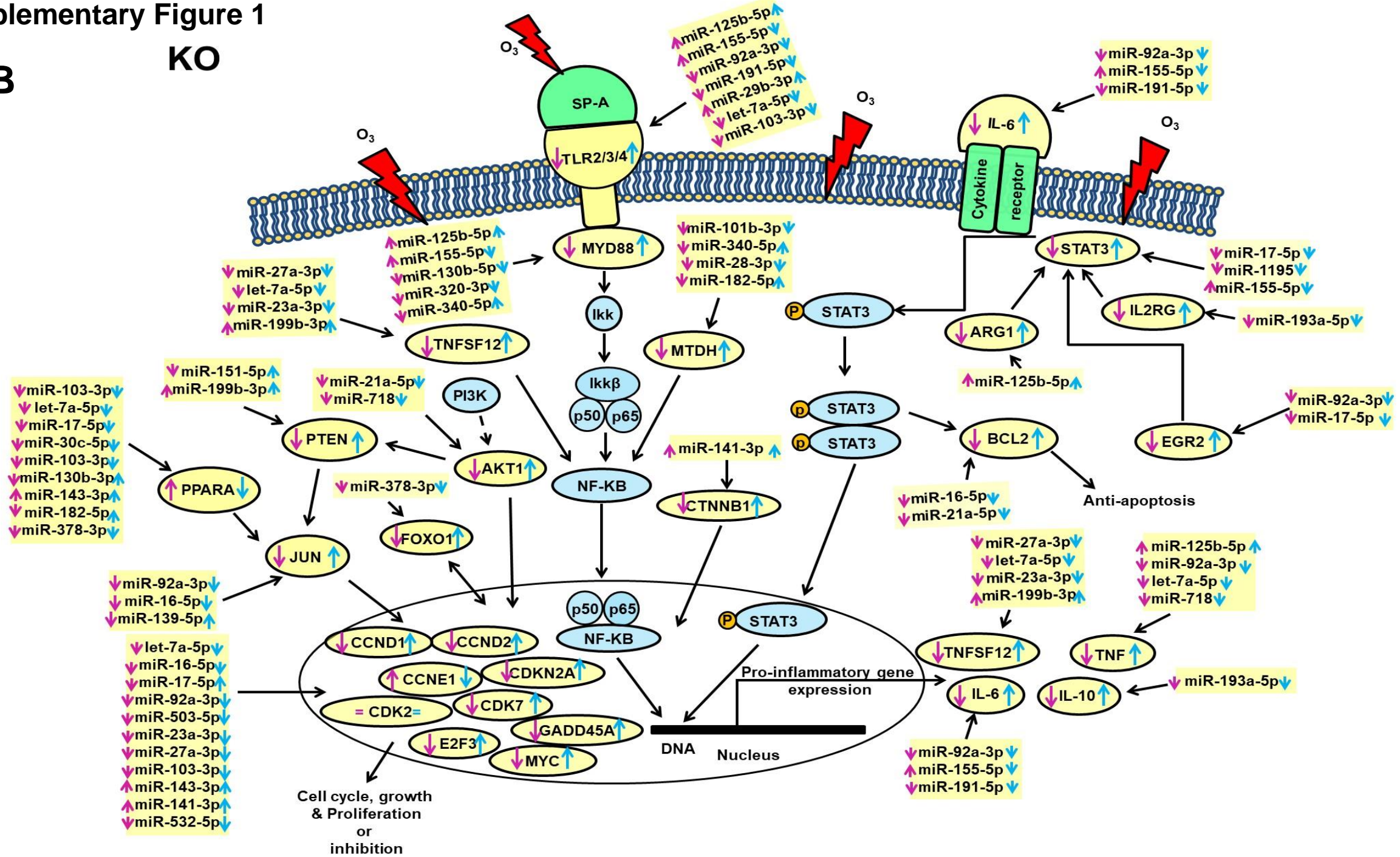

Supplement: Supplementary file 1 [file antioxidants-09-01190-s001.zip › Supplementary Figure 1A & B.pdf]
